# Efficacy of ascorbic acid, citric acid and *Thevetia* sp. extract against *Agrobacterium tumefaciens* and *Meloidogyne incognita* infecting guava

**DOI:** 10.1038/s41598-025-21658-3

**Published:** 2025-10-10

**Authors:** Rasha E. Selim, Hanan F. B. Youssef, Mohamed S. Khalil

**Affiliations:** 1 Central Agricultural Pesticides Laboratory (CAPL), Agricultural Research Center El- Sabaheya, Alexandria, Egypt; 2 Bacterial Diseases Research Department, Plant Pathology Research Institute, Agricultural Research Center, El- Sabaheya, Alexandria, Egypt

**Keywords:** Guava (*Psidium Guajava* L.), Ascorbic acid, Citric acid, *Thevetia* sp., Agrobacterium tumefaciens, Root-knot nematodes and 16 s rRNA, Plant sciences, Zoology

## Abstract

The efficiency of some organic acids (ascorbic and citric acids) and methanolic extract of *Thevetia* sp. were investigated against crown gall bacterium (*Agrobacterium tumefaciens*) and the root-knot nematode (*Meloidogyne incognita*) under in vitro and pot trails. Two isolates (Ag1 and Ag2) of crown gall bacteria were used in this study. The isolate Ag2 was identified molecularly by PCR using universal primers for 16 S rRNA. The PCR products’ DNA sequence, BLAST analysis and Genbank data revealed that the Ag2 isolate belonged to *A. tumefaciens*. According to the phylogenetic tree based on the DNA nucleotide sequences of the 16 S rRNA gene, the Egyptian isolate is closely similar to the Indian isolate (PP218089). The analysis of *Thevetia* sp. methanolic extract exhibited that 35 phytochemical components were identified by using GC-MS. Under in vitro conditions, ascorbic acid at 6.4% was the most effective against *A. tumefaciens* (Ag2 isolate) recording inhibition zone of 38.3 mm with disc diffusion method and 100% inhibition with MIC method at 1.00 g/l. However, *Thevetia* sp. extract was the most effective against 2nd stage juveniles of *M. incognita* after 48 h of exposure with values of 0.478 g/l (LC_50_) and 3.185 g/l (LC_90_). In pots trial, ascorbic acid 2 (14.8 g/l) completely suppressed both the number and weight of galls (tumors) in guava seedlings subjected to either single or dual infections of *A. tumefaciens* and *M. incognita*. *Thevetia* sp. 2 (6.4 g/l) and ascorbic acid 2 (14.8 g/l) significantly reduced root gall formation and soil populations by 63.40 and 70.27% under single infection of *M. incognita* and 81.67 and 76.57%, under dual infection of *A. tumefaciens* + *M. incognita*, respectively. Furthermore, these treatments markedly enhanced the growth performance of guava seedlings, as evidenced by increased shoot height and root length and significantly elevated the levels of total phenols and total soluble proteins under both single or dual infection scenarios.

## Introduction

Guava (*Psidium guajava* L.) is a tropical fruit tree that is widely cultivated in tropical and subtropical regions due to its adaptability to various soil conditions and its high nutritional value. Guava fruits are main source of lycopene, ß-acarotenes, proteins, fats, carbohydrates, fibers, minerals, vitamins A, B & C^1^. In Egypt guava cultivation is primarily concentrated in the Nile Delta and Upper Egypt regions. In 2022, Egypt cultivated approximately 42,170 feddan of guava, representing 2.4% of the total fruit cultivated area in the country. The production reached 365,556 tons, accounting for 3% of the total fruit production in Egypt. Behira governorate is the top guava producer, contributing around 43% of the national production^2^.

In Egypt, the most famous and devastating pests which attacking guava trees and cause great economic losses, are the plant parasitic nematodes especially the root-knot nematodes (*Meloidogyne* spp.) and the crown gall disease that caused by *Agrobacterium tumefaciens*. Crown gall disease is a major bacterial disease in nurseries and orchards and is being considered one of the most important disorders that causes high losses in nurseries^3^. *A. tumefaciens*, a soil-borne, saprophytic plant pathogen enters plant tissues through wounds and causes infection producing tumors on a broad range of plants including many dicots, few monocots and some gymnosperms^4^. Plant parasitic nematodes (PPNs) caused losses $2.30 billion annually in Egyptian production^5^. The interrelation between nematode densities and increasing of crown gall on the roots was reported for many crops references. Occurrence of wounds by nematodes allows incorporation of T DNA (the transferred DNA of the tumor-inducing (Ti) plasmid) of bacteria into the genome of plant cell and development of crown gall disease^6^. There are many attempts to stop the deadly effect of dual infections of crown gall disease and root-knot nematodes in Egyptian orchard. Using of organic acids is one of the attempts which targeted to manage the *A. tumefaciens* and *M. incognita* on guava trees. Organic acids such as; lactic acid, acetic acid, citric acid and ascorbic acid have been studied for its properties against various plant pathogens. Citric or ascorbic acids recorded nematicidal potential against *Meloidogyne* sp^7^^[,8^. and bactericidal activity against *A. tumefaciens*^9,10^.

On the other hand, *Thevetia* sp. belongs to Apocynaceae family and it is commonly known as yellow oleander. It is an ornamental plant which grows in India, China, Australia and different parts of Africa^11^. It has also been regarded as a potential source of insecticides, rodenticides, fungicides and bactericides^12^. Various extracts of *Thevetia* sp. are contain terpenoids, phenols, flavonoids, anthraquinones and free amino acids, whose toxic against a panel of microorganisms such as *Bacillus subtilis*, *Escherichia coli*, *Agrobacterium tumefaciens*,* Xanthomonas phaseoli* and *Erwinia chrysanthemi*^13^. No commercial products are provided till now to control *A. tumefaciens*. Therefore, in this investigation we aimed to study the bacterial and nematicidal impacts of ascorbic acid, citric acid and the methanolic extract of *Thevetia* sp. against *Agrobacterium tumefaciens* and *Meloidogyne incognita* on guava seedlings under single or dual infections. Additionally, to record their effect on the growth indices and the levels of total phenols and soluble proteins in guava seedlings.

## Materials and methods

### Bacterial isolation and culture media

Isolation trials were carried out from infected Peach (*Prunus persica*) and Guava (*Pisidum guajava* L.) trees showing crown gall disease symptoms, collected from Edkou farms and Rashid, El-Beheira governorate, Egypt respectively. The bacteria were isolated from symptomatic materials and streaked on glycerol nutrient agar medium according to a described method^14^. After 48 h of incubation at 28 °C the colonies were observed, isolated, and kept purified on 2% glycerol nutrient agar slants for later use.

### The source of the root knot nematode (*Meloidogyne incognita*) inoculation

The inoculum of the root-knot nematode; *Meloidogyne incognita* was isolated from root of infected eggplant (*Solanum melongena* cv. Black Beauty). The perineal patterns technique was utilized to identify the population of root-knot nematode as *Meloidogyne incognita*, according to^15^. The eggs of root-knot nematodes were extracted by Sodium hypochlorite (NaOCl) method^16^ from infected roots and use 200 and 400 mesh sieves to obtain the free eggs. The second stage juveniles (J_2_) obtained by using the Baermann plate technique^17^.

### Plant leaves collection and extract Preparation

The fresh fruits of *Thevetia* sp. were collected randomly from plants in the garden of integrated protection laboratory, plant protection research station at El-Sabaheya, Alexandria, Egypt. The mature fruits were properly washed with tap water and then rinsed with distilled water. 100 gram of mature fruits were frozen in liquid nitrogen, ground in mortar, then The powdered plant material (100 g was extracted with 500 ml in 1 L of conical flask and covered with aluminum foil paper for 30 days at room temperature with continuous shaking on a shaker, The extracts were then filtered and evaporated on a water bath at low temp. (40–50 °C). the methanol dry extract weighed 15 gm. The extracts so obtained were stored at 4 °C until used^18^.

### Phenotypic identification

The morphological traits of the bacterial isolates were examined by light microscopy. The physiological and biochemical characteristics were carried out according to protocols described by^19^.

### Sequencing of 16 S rRNA gene and alignment

Full length (1550 bp) of 16 S rRNA gene was amplified from the isolate of *Agrobacterium* sp. (Ag2), using the universal primers P0 (F) (5’GAAGAGTTTGATCCTGGCTCAG3’) and P6 (R) (5’CTACGGCTACCTTGTGTTACGA3’). The amplified product of 16 S rRNA gene (1550 bp) was sequenced using a Big Dye Terminator Cycle Sequencing Kit and resolved on an ABI PRISM model 310 Automated DNA Sequencer at the Sigma Scientific Services Company. Pair-wise and multiple DNA sequence alignment were carried out using CLUSTALW (1.82) http://www.ebi.ac.uk/clustalw^20^. Bootstrap neighbor-joining tree generated using MEGA (version 11)^21^ from CLUSTALW alignment. Comparisons with sequences in the GenBank database were achieved in BLASTN searches at the National Center for Biotechnology Information site (http://www.ncbi.nlm.nih.gov).

### Antibacterial activity of organic acids and methanolic plant extract under in vitro conditions by disc diffusion and minimum inhibitory concentration (MIC) methods

The inhibitory effect of ascorbic acid, citric acid and *Thevetia* sp. methanolic extract were assessed under in vitro conditions on the growth of *Agrobacterium* sp. isolates at the concentrations of 4, 8, 16, 32, 45 and 64 g/l. by agar disc diffusion method according to^22^. In respect to minimum inhibitory concentration (MIC) appropriate volumes of the stock solutions (0.1, 0.2, 0.25, 0.30, 0.5, 1, 1.5, 1.75, 2.0, 2.5, 3.0, 3.5 and 4 g/l) recommended by European Society of Clinical Microbiology and infection Disease^23^. However, the evaluated treatments were dissolved in distilled water contains dimethyl sulfoxide (DMSO) at 0.5%, and tween 20 at 0.1%^24^. Each treatment was replicated four times and replicates included distilled water served as a control, whilst replicates contain DMSO and tween 20 served as a blank.

### The activity of two organic acids and methanolic plant extract against 2nd stage juveniles of *Meloidogyne incognita* under in vitro conditions

Assessment of the effect of ascorbic acid, citric acid and the methanolic extract of *Thevetia* sp. on the mortality of *M*. *incognita* J_2_ was carried out in vitro. Ascorbic and citric acids were tested at 0.125, 0.25, 0.5, 1.00, 2.00, 3.00 and 6.00 g/l. Moreover, the methanolic extract of *Thevetia* sp. was tested at 0.0625, 0.125, 0.25, 0.50, 1.00, 2.00 and 4.00 g/l. Each treatment was replicated four times and each replicate (vial ca. 10 ml) involved 200 J_2_. Replicates included distilled water served as control, whilst replicates contain dimethyl sulfoxide (DMSO) at 0.5%, and tween 20 at 0.1% served as blank^24^. After exposure for 48 h, the numbers of both dead and alive J_2_ were recorded and the mortality percentages was calculated.

### Gas chromatography–mass spectrometry (GC-MS) analysis

Using a direct capillary column TG–5MS (30 m x 0.25 mm x 0.25 μm film thickness), the Trace GC-TSQ mass spectrometer (Thermo Scientific, Austin, TX, USA) was used to analyze the chemical composition of the extracts of methanol, methylene chloride, and petroleum ether. The column oven temperature was first maintained at 50 °C, then raised by 5 °C/min to 250 °C and maintained for 2 min. Finally, it was raised by 30 °C/min to the ultimate temperature of 300 °C and maintained for 2 min. The temperatures of the injector and MS transfer line were maintained at 270 and 260 °C, respectively. A steady flow rate of 1 ml/min of helium was employed as the carrier gas. Diluted samples containing 1 µl were automatically injected using an Autosampler AS1300 connected to a GC in split mode, with a 4-minute solvent delay. In full scan mode, EI mass spectra were obtained at 70 eV ionization voltages covering m/z 50–650. A temperature of 200 °C was chosen for the ion source. By comparing the components’ mass spectra with those from the NIST 14 mass spectral database and WILEY 09 mass spectra, the components were identified^25^.

### The pots trial procedures

The bactericidal and nematicidal efficacy of ascorbic acid, citric acid and *Thevetia* sp. extract were investigated against the crown gall disease (*Agrobacterium tumefaciens*), the root-knot nematode (*M. incognita*) and their dual infection. The applied concentrations of ascorbic acid, citric acid and *Thevetia* sp. extract were the values of LC_90_ (7.4, 5.8 and 3.2 g/l) and their fold (14.8, 11.6 and 6.4 g/l) from the in vitro assay against the 2nd stage juveniles of *M. incognita* after 48 h of exposure. The organic acids namely; ascorbic and citric acids were obtained from El-Nasr chemicals company, Alexandria, Egypt. The nematicide; Oxamytod^®^ 24% SL (Oxamyl) applied at the rate of 4 L/feddan for comparison. The experiment was conducted at the plant protection research station of El-Sabaheya, Alexandria. The used guava seedling (Local variety “Banaty”) were nine months old. All treatments were run 24 h after inoculation. Each treatment was replicated five times. Also, each pot (replicate) was received 200 ml of solution’s treatment. Pots treated with sterile distilled water were used as untreated uninfected control. While seedlings infected with *A. tumefaciens*,* M. incognita* and their blend served as untreated controls. The guava seedlings were pricked at the crown region and inoculated with ca. 5 × 10^8^ CFU/ml of the selected *A. tumefaciens* (Ag2) isolate. The inoculation with the second stage juveniles of the root-knot nematode (*M. incognita*) was at 2000 individuals/seedling. The dual infection was done by using both mentioned inoculations. Irrigation and fertilization were done when needed. The used pots were 21 cm diameter which filled with approximately 3 kg of autoclaved loamy sand soil (pH = 8, OM = 0.7%, sand: silt: clay = 82:10:8%). In the termination of the experiment about 52 days of inoculations the seedlings were uprooted gently and washed to estimate the number of galls/root system and J_2_/Kg soil. The weights (g) and numbers of galls caused by *A. tumefaciens* were recorded with single or dual infections. The reduction percentages in weight and number of galls of crown gall disease was calculated as follows^26^:

$$\:Reduction\text{\%}=\left(\frac{Control-Treatment}{Control}\right)\times\:100$$

where C = weight or number of galls in control and T = weight or number of galls in treatment. The shoot height and root length of guava seedling, in addition to total phenol and total soluble proteins were assessed.

### The applied treatments during the pot trial

- Ascorbic acid at 7.4, and 14.8 g/l + *A. tumefaciens*.
- Ascorbic acid at 7.4, and 14.8 g/l + *M. incognita*.
- Ascorbic acid at 7.4, and 14.8 g/l + *A. tumefaciens* + *M. incognita*.
- Citric acid at 5.8 and 11.6 g/l + *A. tumefaciens*.
- Citric acid at 5.8 and 11.6 g/l + *M. incognita*.
- Citric acid at 5.8 and 11.6 g/l + *A. tumefaciens* + *M. incognita*.
- Methanolic extract of *Thevetia* sp. at 3.2 and 6.4 g/l + *A. tumefaciens*.
- Methanolic extract of *Thevetia* sp. at 3.2 and 6.4 g/l + *M. incognita*.
- Methanolic extract of *Thevetia* sp. at 3.2 and 6.4 g/l + *A. tumefaciens* + *M. incognita*.
- Oxamyl at 0.012 ml/replicate.
- Untreated uninfected control.
- Untreated control + *A. tumefaciens*.
- Untreated control + *M. incognita*.
- Untreated control + *A. tumefaciens + M. incognita*.

### Assessment of the total phenol content

Total soluble phenol contents in guava leaves, was extracted according to^27^, the absorbance at 765 nm was determined by using Spectrophotometer (Tuner, model 390). Total soluble phenol content was standardized against tannic acid and absorbance values were converted to mg of phenols/g fresh weight of leaves. Each value reported was the average of five replicates. The results were expressed as tannic equivalent according to this formula.

mg tannic acid/g fresh weight = (((OD/K) * (10/0.2)/0.5)).

Where: OD = absorbance at 765 nm, K = the extension coefficient = 0.016898 µg-¹.

### Assessment of the total soluble protein

Total protein was extracted according to^28^^[,29^. The developed blue color was measured at 700 nm by using Spectrophotometer (Tuner, model 390). The reading was related to standard curve prepared from known concentration of BSA protein.

µg protein/g fresh weight = (((OD/K) * 100)/0. 5)/1000)

Where: OD = The absorption at 700 nm, K = 0.0383.

### Statistical analysis

The statistical analysis of data was carried out using the computer program^30^. The experimental units were arranged in a complete randomized design (CRD) with five replications for each treatment, each replicate consisted of one seedling. Statistically, the significant differences between the means were compared using analysis of variance (ANOVA) with the least significant differences (LSD) and *P* values = 0.05 probability. The mortality percentages of 2nd stage juveniles were estimated using^31^, and the Probit analysis was used to calculate LC_50_ and LC_90_ for each compound according to^32^.

## Results

### The bacterial isolation and initial characterizations

Attempts to isolate *Agrobacterium* sp. from twenty-five guava root gall samples resulted in two different isolates coded Ag1 isolated from Edkou farms and Ag2 isolated from Rashid farms. After 48 h from incubation at 28 °C, distinct colonies that had typical morphological and biochemical traits of *Agrobacterium tumefaciens* were purified and listed in (Table 1).

**Table 1:** Morphological, physiological and biochemical reactions traits of *Agrobacterium tumefaciens* isolates.

Characteristics		Bacterial Isolates	
		Ag1	Ag2
Shape(rods)		+	+
Gram staining		-	-
Motility		+	+
Anaerobic growth		+	+
Mucoid growth		+	+
Growth in 5% NaCl		-	-
Indole production		-	-
Oxidase reaction		+	+
Growth at 37 °C		+	+
Catalase test		+	+
Acid production	Lactose Sucrose Mannose Fructose Maltose Glucose Manitol	A A - - - - A	A A - - - - A
Urease production		-	-

(+) = Positive reaction, (-) = Negative reaction and A= acid

#### The bacterial 16 s rRNA partial gene identification

The region of the 16 S rRNA gene (1550 bp) was amplified for Ag2 bacterial isolate. A partial sequence for *Agrobacterium* sp. isolate Ag2 was acquired when a large (1550 bp) purified portion of the 16 s rRNA gene was sequenced using an automated DNA sequencer, the ABI PRISM model 310 at Sigma Company. The BLAST search (http://www.ncbi.nlm.nih.gov) revealed that the sequence corresponding to the bacterial isolate Ag2 was almost identical to that of *Agrobacterium tumefaciens* (Syn, *Rhizobium radiobacter*). The homology of the Egyptian *A. tumefaciens* isolate to the GenBank strains reached 100%. The sequence was submitted to GenBank with accession number of PP646500.

### Phylogenetic analysis

**Fig. 1:**
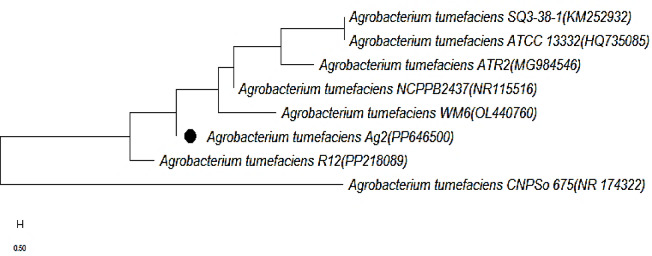
Phylogenetic tree constructed upon bootstrap neighbor-joining tree method based on 16 s rRNA gene partial sequence of *Agrobacterium tumefaciens* (Ag2) isolate.

Alignment of 16 s rRNA sequence of *Agrobacterium tumefaciens* (Ag2) isolate with the 16 s rRNA sequence of other *Agrobacterium* strains collected from the GenBank was carried out using CLUSTAL W (1.82) (http://www2.ebi.ac.uk/clustalw)^20^ from which MEGA (version 11)^21^ was used to generate the Bootstrap neighbor-joining tree (Fig. 1). The phylogenetic tree constructed based on the DNA nucleotide sequences of the 16 s rRNA gene revealed that the Egyptian isolate (Ag2) is closely related to Indian isolate (PP218089) with similarity 96%. Furthermore, the Egyptian isolate (Ag2) is related to the Chinese isolate (OL440760) and the Spanish isolate (HQ735085) with similarity 92%.

### The bactericidal activity of organic acids and *Thevetia sp*. extract with disc diffusion method

The bactericidal activity of ascorbic and citric acids, in addition to *Thevetia* sp extract were assessed on the growth of two bacterial isolates of *A. tumefaciens* (Ag1and Ag2) by using the disc diffusion method under in vitro conditions at concentrations of 4, 8, 16, 32, 45 and 64 g/l (Table 2). The obtained results revealed that the *A. tumefaciens* (Ag1) recorded inhibition zones with ascorbic acid (ranged from 0.3 to 23.3 mm), citric acid (1.7 to 13.7 mm), and *Thevetia* sp. extract (0.7 to 9.0 mm). Similarly, the Ag2 isolate showed inhibition zones with ascorbic acid (ranged from 7.3 to 38.3 mm), followed by citric acid (2.7 to 24.7 mm) and *Thevetia* sp. extract (1.0 to 18.0 mm). The most effective treatment was ascorbic acid followed by citric acid and *Thevetia* sp. extract. Both tested isolates (Ag1and Ag2) were sensitive to ascorbic acid, citric acid and *Thevetia* sp. extract. No significant differences were noticed between Ag1 and Ag2 isolates. The most effective concentration was 64 g/l. Also, ascorbic acid treatment at various concentrations recorded significant differences with Ag2 isolate of *A. tumefaciens*.

**Table 2:** The bactericidal activity ascorbic acid, citric acid and *Thevetia* sp. extract. against *Agrobacterium tumefaciens* isolates using disc diffusion method.

**Treatments**	**Conc. (g/l)**	**Inhibition zone (mm)**		**The main effect of concentrations**	**The main effect of treatments**
		** Ag1**	** Ag2**		
Ascorbic acid	4	0.3h	7.3ij	4 g/l (0.23b)	1.47A
	8	1.7gh	11.3g		
	16	4.3def	16.3ef		
	32	9.0c	21.7d	8 g/l(0.41b)	
	45	13.3b	29.0b		
	64	23.3a	38.3a		
Citric acid	4	1.7gh	2.7mn	16 g/l (0.61b)	0.82AB
	8	3.0fg	4.3klm		
	16	4.0ef	6.7ijk		
	32	5.3de	8.7hi	32 g/l (0.88b)	
	45	8.7c	14.3f		
	64	13.7b	24.7c		
*Thevetia* sp.	4	0.7h	1.0no	45g/l (1.37b)	0.53BC
	8	1.3gh	2.7mn		
	16	1.7gh	3.7lm		
	32	3.0fg	5.3jkl	64 g/l (4.34a)	
	45	6.0d	11.0gh		
	64	9.0c	18.0e		
Control	---	0.00h	0.00o	----	0.00C
The main effect of bacterial isolates		0.61A	1.26A	----	----

Means in each column or row followed with the same letter are not significantly different according to LSD test (*P*< 0.05).

### The bactericidal activity of ascorbic acid, citric acid and *Thevetia* sp. extract by minimum inhibitory concentration (MIC) method

The impact of ascorbic acid, citric acid and *Thevetia* sp. extract at different concentrations ranging from 0.10 to 4.00 g/l on the growth of two bacterial isolates of *A. tumefaciens* (Ag1 and Ag2) by using the minimum inhibitory concentration (MIC) were illustrated in Table (3). All tested concentrations exhibited varying degrees of inhibition activity on the bacterial strains. The MICs of ascorbic acid, citric acid and *Thevetia* sp. extract were 3.50, 4.00 and > 4.00 g/l which completely inhibited the Ag1 isolate. Meanwhile, the MICs for Ag2 were 1.00, 4.00 and > 4.00 g/l with ascorbic acid, citric acid and *Thevetia* sp. extract resulting in complete inhibition, respectively.

**Table 3:** Antibacterial activity of ascorbic acid, citric acid and *Thevetia* sp. extractagainst *Agrobacterium tumefaciens* isolates using minimum inhibitory concentration (MIC) method.

**Treatments**	**Bacterial isolates (MIC** **g/l**	
	**Ag1**	**Ag2**
Ascorbic acid	3.50	1.00
Citric acid	4.00	4.00
*Thevetia* sp. extract	>4.00	>4.00

### The effectiveness of ascorbic acid, citric acid and the methanolic extract of *Thevetia* sp. on the larvae of the root-knot nematodes (*M. incognita*) under *in vitro* conditions

The larvicidal activity of ascorbic acid, citric acid and the methanolic extract of *Thevetia* sp. were evaluated at various concentrations against the second-stage juveniles (J_2_) of the root-knot nematode (*M. incognita*) after 48 h of exposure (Table 4). Ascorbic and citric acids were assessed at 0.25, 0.50, 1.00, 2.00, 4.00 and 6.00 g/l, while the methanolic extract was evaluated at 0.125, 0.25, 0.50, 1.00, 2.00 and 4.00 g/l. Based on the obtained results the methanolic extract was the most toxic treatment followed by citric and ascorbic acids to the larvae of the root-knot nematode (*M. incognita*) with calculated LC_50_ values of 0.478, 0.857 and 0.966 g/l, respectively. The same trend was noticed with LC_90_ values of 3.185, 5.821 and 7.368 g/l with treatment of methanolic extract, citric and ascorbic acids, respectively. Generally, it could be arranging treatments in a descending order according to their activity (toxicity) on larval mortality as follows: the methanolic extract of *Thevetia* sp. ˃ citric acid ˃ ascorbic acid.

**Table 4:** The LC_50_, LC_90_ and slope values of ascorbic acid, citric acid and *Thevetia* sp. extract on the 2^nd^ stage juveniles of *Meloidogyne incognita*, after 48 h of exposure.

Treatments	LC_50_(g/l/48 h) (Lower-Upper)	LC_90_g/l/48 h) (Lower-Upper)	Slope ± variance
Ascorbic acid	0.966 (0.8161–1.141.8161.141)	7.368 (5.521–10.622.521.622)	1.45 ± 0.10
Citric acid	0.857 (0.729–1.005.729.005)	5.821 (4.481–8.076.481.076)	1.54 ± 0.11
*Thevetia *sp.	0.478 (0.407–0.561.407.561)	3.185 (2.447–4.426.447.426)	1.56 ± 0.10

### Effect of ascorbic acid, citric acid and *Thevetia* sp. extract on the incidence of crown gall on guava seedlings

**Fig. 2:**
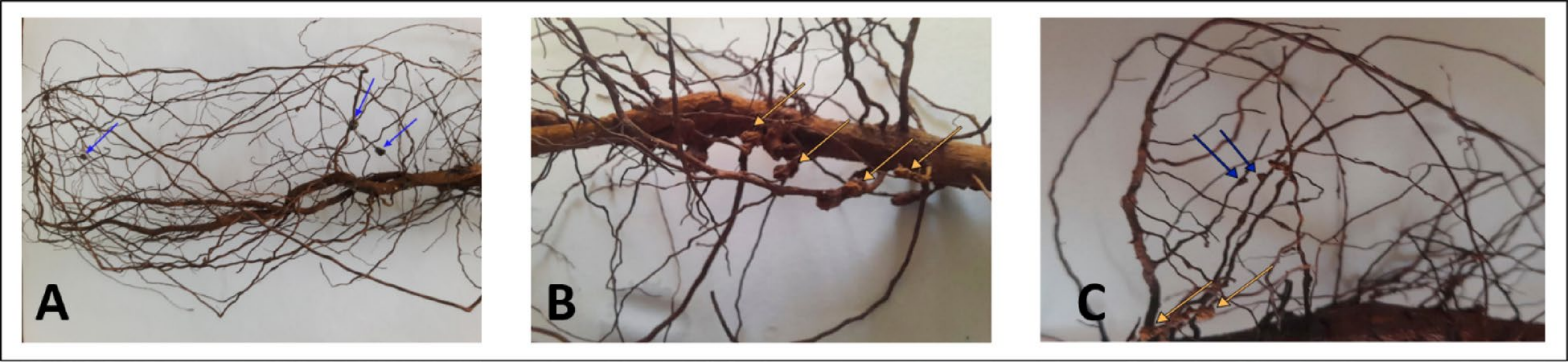
Tumors induced by *Agrobacterium tumefaciens* marked by blue arrows (**A**), galls induced by *Meloidogyne incognita* marked by yellow arrows (**B**), and induced tumors (blue arrows) by *A. tumefaciens *and galls by *M. incognita* (yellow arrows) in dual infection (**C**) on the roots of guava seedlings.

The impacts of ascorbic acid, citric acid and *Thevetia* sp. extract against the crown gall disease that caused by the selected *A. tumefaciens* (Ag2) isolate on guava seedlings in the absence or presence of *M. incognita* were shown in Table (5) and Fig. (2). The obtained results with single infection (*A. tumefaciens*) exhibited that the number of bacterial crown galls (tumors) were decreased significantly within a range of 13.04 to 100.00%, whilst galls weight reduced within a range of 16.67 to 100.00%. However, in dual infection of *A. tumefaciens* + *M. incognita* the number of bacterial crown galls were decreased within a range of 10.48 to 100.00%, and galls weight reduced within a range of 20.63 to 100.00%. Ascorbic acid 2 was the superior treatment which suppressed gall numbers and weights in both infection cases by 100%. There were clear significant differences between the two rates (1&2) of applied treatments in numbers and weights of galls with the single infection, whilst this significance was disappeared with the dual infections for the same rates of applied treatments in galls weight.

**Table 5:** Activity of ascorbic acid, citric acid and *Thevetia* sp. extract on the numbers and weights of crown galls of *Agrobacterium tumefaciens* alone or along with *Meloidogyne incognita* on guava seedling.

Treatments	*A. tumefaciens*(Ag2)				*A. tumefaciens *+ *M. incognita*			
	No. of galls/plant	R %	Galls weight/plant (g)	R %	No. of galls/plant	R %	Galls weight/plant (g)	R %
Ascorbic acid 1	1.33e	82.61	0.05e	89.33	1.00d	84.20	0.04cd	90.48
Ascorbic acid 2	0.00f	100.00	0.00f	100.00	0.00e	100.00	0.00d	100.00
Citric acid 1	3.33d	56.52	0.18d	63.33	2.33c	63.14	0.09c	77.78
Citric acid 2	1.67e	78.26	0.09e	82.00	1.33d	78.94	0.05cd	88.10
*Thevetia* sp. 1	6.67b	13.04	0.42b	16.67	5.67ab	10.48	0.33b	20.63
Thevetia sp. 2	4.33c	43.48	0.23c	53.33	5.33b	15.75	0.32b	24.60
Untreated control	7.67a	0.0	0.50a	0.0	6.33a	0.0	0.42a	0.0

Means in each column followed with the same letter are not significantly different according to LSD test (*P*< 0.05). R%= Reduction percentage. Ascorbic acid 1 = 7.4 g/l (LC_90_ value), Ascorbic acid 2 = 14.8 g/l (one fold of LC_90_ value), Citric acid 1 = 5.8 g/l, Citric acid 2 = 11.6 g/l,* Thevetia* sp. 1 = 3.2 g/l, *Thevetia* sp. 2 = 6.4 g/l.

### The performance of ascorbic acid, citric acid and *Thevetia* sp. extract against the root-knot nematode (*M. incognita*) alone or in blend with *Agrobacterium tumefaciens*

The nematicidal activity of ascorbic acid, citric acid and the methanolic extract of *Thevetia* sp. was assessed against the root-knot nematode (*M. incognita*) alone or in the presence of *A. tumefaciens* on the root of guava seedlings in a pot trial (Table 6; Fig. 2). Oxamyl treatment suppressed the root galls and soil populations by 67.97 and 84.92%, consecutively. The root galls were reduced significantly by 36.60 and 52.94% with ascorbic acid 1&2, while recorded 31.37 and 42.48% with citric acid 1&2, respectively. Furthermore, the highest reductions in root galls were recoded with *Thevetia* sp. extract 1&2 by 47.06 and 63.40%, respectively. Otherwise, the soil population were decreased by 65.83 and 81.67%, 24.27 and 67.8%, and 43.26 and 61.37% with ascorbic acid 1&2, citric acid 1&2 and *Thevetia* sp. extract 1&2, respectively. On the other hand, in the presence of the bacterium; *A. tumefaciens*, soil treated with oxamyl gave reductions in guava root galls and soil populations by 78.38 and 83.05%, respectively. The obtained results showed that applied treatments recorded reductions in root galls by 67.57 and 64.33%, 61.08 and 65.41% & 47.48 and 68.34% with ascorbic acid 1&2, citric acid 1&2 and *Thevetia* sp. extract 1&2, respectively. Also, the soil populations were minimized significantly with ascorbic acid 1&2 (59.75 and 76.57%), citric acid 1&2 (13.84 and 60.23%) and *Thevetia* sp. extract 1&2 (47.48 and 68.34%). Significant differences were noticed between the two rates (1&2) of applied treatments in galls/root system and soil population (J2/kg soil) with single infection (*M. incognita*). Meanwhile, the dual infection of *A. tumefaciens* + *M. incognita* exhibited significant differences between the two rates (1&2) of applied treatments in soil population (J2/kg soil), while this significance was disappeared with the same rate of applied treatments in galls/root system.

**Table 6:** The effect of ascorbic acid, citric acid and *Thevetia *sp*.* extract on galls and J_2_ of *Meloidogyne incognita* alone or in blend with *Agrobacterium tumefaciens*.

Treatments	*M. incognita*				*A. tumefaciens *+ *M. incognita*			
	Galls/root system	R%	J_2_/kg soil	R%	Galls/root system	R%	J_2_/kg soil	R%
Ascorbic acid 1	32.33bc	36.60	730.33de	65.83	20.00bc	67.57	957.00d	59.75
Ascorbic acid 2	24.00de	52.94	391.67f	81.67	22.00b	64.33	557.00f	76.57
Citric acid 1	35.00b	31.37	1618.67b	24.27	24.00b	61.08	2048.67b	13.84
Citric acid 2	29.33bcd	42.48	703.67e	67.08	21.33b	65.41	945.67d	60.23
*Thevetia* sp. 1	27.00cd	47.06	1212.67c	43.26	22.33b	63.79	1248.67c	47.48
*Thevetia* sp. 2	18.67ef	63.40	825.67d	61.37	18.33bc	70.27	752.67e	68.34
Oxamyl	16.33f	67.97	322.33f	84.92	13.33c	78.38	403.00g	83.05
Untreated control	51.00a	---	2137.33a	---	61.67a	---	2377.67a	---

Means in each column followed with the same letter are not significantly different according to LSD test (*P*< 0.05). R%= Reduction percentage. Ascorbic acid 1 = 7.4 g/l (LC_90_ value), Ascorbic acid 2 = 14.8 g/l (one fold of LC_90_ value), Citric acid 1 = 5.8 g/l, Citric acid 2 = 11.6 g/l,* Thevetia* sp. 1 = 3.2 g/l, *Thevetia* sp. 2 = 6.4 g/l.

**Table 7:** Effect of ascorbic acid, citric acid and *Thevetia* sp. extract on the growth parameters of guava seedling infected with * Agrobacterium tumefaciens* and *Meloidogyne incognita* alone or in blend.

Treatments	*Meloidogyne incognita*				*A. tumefaciens*				*A. tumefaciens* + *M. incognita*			
	Shoot system height		Root system Length		Shoot system height		Root system Length		Shoot system height		Root system length	
	Mean	I %	Mean	I %	Mean	I %	Mean	I %	Mean	I %	Mean	I %
Ascorbic acid 1	104.00ab	17.95	44.33bc	29.32	99.00ab	9.09	44.00bc	15.91	94.00bc	17.02	36.67bcd	14.55
Ascorbic acid 2	85.67d	0.39	42.83bc	26.85	99.33ab	9.40	45.00b	17.78	99.33ab	21.48	40.00b	21.68
Citric acid 1	90.00cd	5.19	41.00c	23.58	97.67ab	7.85	40.33bcd	8.26	88.00bcd	11.36	36.00bcd	12.97
Citric acid 2	108.33a	21.23	40.00c	21.67	83.00b	−8.43	33.67de	−9.90	75.67d	−3.08	39.33bc	20.35
*Thevetia* sp. 1	95.67bc	10.80	44.33bc	29.32	97.33ab	7.53	32.33e	−14.43	92.33bc	15.52	30.67d	−2.16
*Thevetia* sp. 2	103.67ab	17.68	38.33c	18.26	95.33ab	5.59	33.67de	−9.90	82.00cd	4.88	34.00bcd	7.85
Oxamyl	106.00a	19.50	49.33ab	36.49	---	---	---	---	96.00ab	18.75	35.33bcd	11.33
Untreated uninfected control	107.33a	20.50	53.33a	41.25	107.33a	16.15	53.33a	30.63	107.33a	27.33	53.33a	41.26
**Untreated control**	**85.33d**	**---**	**31.33d**	**---**	**90.00ab**	**---**	**37.00cde**	**---**	**78.00d**	**---**	**31.33cd**	**---**

Means in each column followed with the same letter are not significantly different according to LSD test (*P*< 0.05). I %= increase percent, Ascorbic acid 1 = 7.4 g/l (LC_90_ value), Ascorbic acid 2 = 14.8 g/l (one fold of LC_90_ value), Citric acid 1 = 5.8 g/l, Citric acid 2 = 11.6 g/l,* Thevetia* sp. 1 = 3.2 g/l, *Thevetia* sp. 2 = 6.4 g/l.

### Effectiveness of ascorbic acid, citric acid and *Thevetia* sp. methanolic extract on the growth of guava seedlings

The impact of applied treatments namely; ascorbic acid, citric acid and the methanolic extract of *Thevetia* sp. was investigated on the growth characterization of guava seedlings infected with the root-knot nematode (*M. incognita*) and/or the crown gall disease, *Agrobacterium tumefaciens*, in addition to their blend (Table 7). Oxamyl treatment showed an increase in guava shoot height and root length by 19.50 and 36.49%, respectively, in seedlings infected with *M. incognita*. Meanwhile, shoot height and root length of seedlings infected with *A. tumefaciens* + *M. incognita* were increased by 18.75 and 11.33% with oxamyl treatment, respectively. Regarding the untreated uninfected control exhibited increments in shoot height and root length by 20.50 & 41.25%, 16.15 & 30.63% and 27.33 & 41.26% in guava seedlings infected with *M. incognita*,* A. tumefaciens* + *M. incognita* and *A. tumefaciens*, consecutively.

The shoot system height was relatively increased with all applied treatments within a range of 0.39 to 21.23%, while the root system length was increased insignificance within a range of 18.26 to 29.32% in seedlings infected with *M. incognita* only. Notably fluctuations were observed between the high and low concentrations of treatments in the recorded increment (%) values. In respect to the effect of applied treatments on the seedlings growth infected with *A. tumefaciens*, the results revealed that the shoot system height was increased insignificance within a range of 5.59 to 9.40%, except for citric acid 2 which decreased by 8.43%. Application of ascorbic acid 1 and 2 as well as citric acid 1 were increased the root length within a range of 8.26 to 17.78%, while citric acid 2 and *Thevetia* sp. 1 and 2 were decreased the root length within a range of 9.90 to 14.43%. The shoot height was increased within a range of 4.88 to 21.48% with all applied treatments against the dual infection of *A. tumefaciens* + *M. incognita*, except for citric acid 2 which recorded reduction by 3.08%. However, the root length showed an increase within a range of 7.85 to 21.68%, except for *Thevetia* sp. 1 which recorded reduction by 2.16%.

### The effect of ascorbic acid, citric acid and *Thevetia* sp. extract on the levels of the total phenol and total soluble protein in leaves of guava seedlings

In the presence of *M. incognita*, the level of total phenol and total soluble protein were increased within a range of 29.17 to 56.87 and from 115.53 to 154.46 mg/g in compared with untreated control at 27.64 and 102.35 mg/g, respectively (Table 8). However, the total phenol and total soluble protein under crown gall disease conditions (*A. tumefaciens*) were increased within a range of 47.60 to 58.38 mg/g and from 140.55 to 181.02 mg/g in compared with the untreated control of 34.33 and 135.01 mg/g, respectively. The total phenol and total soluble protein showed increment within a range of 44.25 to 68.45 mg/g and from 146.04 to 294.61 mg/g in compared with the untreated control of 32.17 and 125.31 mg/g, respectively, with the dual infection of *M. incognita + A. tumefaciens*.

### GC–MS analysis of *Thevetia* sp. methanolic extract

**Fig. 3:**
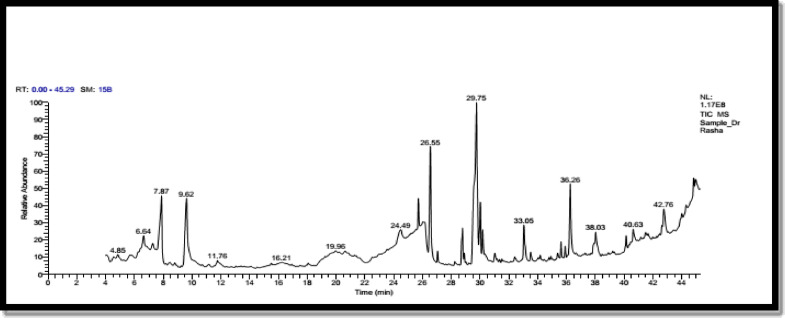
GC–MS spectrum of the phytochemical constituents of *Thevetia* sp. methanolic extract.

The phytochemical components of the methanolic extract were identified by using GC-MS and the chromatogram was shown in Fig. (3). A total of 35 phytochemical components were identified and their names, retention times (RT), peak areas (PA), molecular formulas and molecular weights were reported in Table (9)**.** The phytochemicals have been presented between retention times of 4.85 and 44.3 min. The highest peak areas were achieved with n-Hexadecanoic acid (RT: 26.56 min. and PA: 10.6%), cis-13 Octadecenoic acid (RT: 29.76 min. and PA: 12.3%), 4 H-Pyran-4-one,2,3-dihydro-3,5-dihydroxy − 6-methyl (RT: 7.88 min. and PA: 8.89%), 5- Hydroxymethyl furfural (RT: 9.61 min. and PA: 8.4%) and Erucic acid (RT: 36.27 min. and PA: 7.87%), trilinolein (RT: 44.3 min. and PA: 8.1%), Dodecanoic acid,2,3-bis(acetyloxy) propyl ester (RT: 24.43 min. and PA: 4.33%) and cis-13-Eicosenoic acid (RT: 33.05 min. and PA: 3.68%).

**Table 8:** Effect of ascorbic acid, citric acid and *Thevetia* sp. extract on the levels of the total phenol and total soluble protein in leaves of guava seedlings infected with *Agrobacterium tumefaciens* and *Meloidogyne incognita* alone or in blend.

**Treatments**	*Meloidogyne incognita*		*A. tumefaciens*		*A. tumefaciens* + *M. incognita*	
	Total phenol (mg/g)	Total soluble protein (µg/g)	Total phenol (mg/g)	Total soluble protein (µg/g)	Total phenol (mg/g)	Total soluble protein (µg/g)
**Ascorbic acid 1 **	42.75bc	115.53c	53.60ab	168.91ab	51.87c	146.04d
**Ascorbic acid 2**	43.33b	154.46a	57.53a	181.02a	65.88a	201.89b
**Citric acid 1**	37.51c	130.66b	47.60b	144.21b	47.78cd	143.83d
**Citric acid 2**	29.17d	133.77b	52.33ab	158.02ab	57.47b	210.65b
*Thevetia*** sp. 1**	56.87a	128.11b	57.50a	154.45ab	44.25d	165.43c
*Thevetia*** sp. 2**	43.20b	133.04b	58.38a	140.55b	68.45a	294.61a
**Untreated control**	27.64d	102.35d	34.33c	135.01b	32.17e	125.31e

Means in each column followed with the same letter are not significantly different according to LSD test (*P*< 0.05). Ascorbic acid 1 =7.4 g/l (LC_90_ value), Ascorbic acid 2 = 14.8 g/l (one fold of LC_90_ value), Citric acid 1 = 5.8 g/l, Citric acid 2 = 11.6 g/l,* Thevetia* sp. 1 = 3.2 g/l, *Thevetia* sp. 2 = 6.4 g/l. .

**Table 9:** The phytocompounds identified in the methanolic extracts of *Thevetia* sp. by GC-MS analysis.

RT	Compound name	Molecular weight	Area %
4.85	L-Glucose	180	0.74
6.64	Thymidine	242	1.15
7.27	L-Mannose	180	0.68
7.88	4H-Pyran-4-one,2,3-dihydro-3,5-dihydroxy-6-methyl-	144	8.89
9.61	5-Hydroxymethyl furfural	126	8.4
11.76	4-Pentynet hioamide,N, N-diethyl-2 methyl-	183	0.81
24.43	Dodecanoica cid,2,3-bis(acetyloxy)propyl ester	358	4.33
25.71	Palmitic Acid methyl ester	270	2.24
26.17	Myo-Inositol, 4-C-methyl	194	3.67
26.56	n-Hexadecanoic acid	256	10.6
27.04	Hexadecanoic acid, ethyl ester	284	0.87
28.24	á-D-glucopyranoside,methyl	362	0.44
28.78	Linolenic Acid Methyl Ester	292	3.77
28.90	9,9-Dideutero-octadecanoate	300	0.55
29.54	9,12-Octadecadienoic acid (Z,Z)-	280	2.76
29.76	cis-13-Octadecenoic acid	282	12.3
30.01	Ethyl 9,12,15-octadecatrienoate	306	3.61
30.18	Octadecanoic acid	284	2.13
31.02	Z,Z-3,15-Octadecadien-1-ol acetate	308	0.83
31.15	cis-13-Eicosenoic acid	310	0.28
32.4	Octadecanal, 2-bromo-	346	0.37
33.05	cis-13-Eicosenoic acid	310	3.86
33.51	9-Octadecenoic acid (Z)	282	1.58
35.38	Hexadecanoic acid,2,3dihydroxypropyl ester	330	0.58
35.62	13-Docosenoic acid, methyl ester, (Z)-	352	1.3
35.91	9-(2',2'-Dimethylpropanoilhydrazono)−3,6-dichloro-2,7-bis-[2-(diethylamino)ethoxy] fluorene	576	0.98
36.27	Erucic acid	338	7.87
37.91	9,12-Octadecadienoic acid (Z,Z)-,2,3-dihydroxypropyl ester	354	1.4
38.03	9-Octadecenoic acid (Z)-,2-hydroxy-1-(hydroxymethyl)ethyl ester	356	3.16
39.21	Betulin	442	0.37
40.26	Docosanoic acid,8,9,13-trihydroxy-, methyl ester	402	1.38
44.3	Trilinolein	878	8.1

## Discussion

In various regions of the Beheira Governorate, Egypt, particularly in the Rosetta and Edkou centers, 80% of guava plants are invaded by the bacterial pathogen responsible for crown gall disease. This disease becomes even more hazardous when accompanied with the plant parasitic nematodes, especially the root-knot nematodes; *Meloidogyne* spp^33^. The isolated *Agrobacterium tumefaciens* appeared smooth, round and bright transparent rings in the margins on glycerol nutrient agar. The bacterial cells were rod-shaped in Gram staining as previously described by^34^. The identity of each of the suspected *A. tumefaciens* isolates was determined by the appropriate physiological and biochemical tests, the results were matched with^35–38^. The selected isolates of *A. tumefaciens* have the ability to form tumors on the roots of guava seedlings which confirm their pathogenicity impact as in Fig. 2 (A) and Fig. 2 (C)^39^.

The results of the molecular techniques (16 S rRNA gene sequence) of the isolated guava bacterial isolate revealed that *Agrobacterium tumefaciens* was associated with crown gall disease^40^. The use of 16 S rRNA gene sequences to study bacterial phylogeny and taxonomy as in Fig. (1) in our study has been by far the most common housekeeping genetic marker 16 S rRNA gene was used to identify the tested isolate. One band with the correct predicted molecular length was found in the tested isolate’s results and the examined isolate’s DNA sequences showed that they belonged to *Agrobacterium tumefaciens* which agreed with those obtained by^33,41–43^.

A general conception on the use of organic acids for plant protection against the most dangerous pathogens and pests was provided by^44^. According to our results ascorbic acid was the most effective against *A. tumefaciens* (Ag1 and Ag2) which in agreement with^45^ who reported that ascorbic acid showed high efficacy against *Erwinia carotovora* subsp. *carotovora* (Ecc), *Erwinia carotovora* subsp. *betavasculorum* (Ecb) and *Burkholderia cepacia* (Bc), while citric acid was the least effective against the tested bacteria. Bartz^46^ showed that ascorbic and citric acid significantly reduced soft rot disease caused by *Erwinia carotovora* pv. *carotovora*. Eltabey^47^ cleared that ascorbic acid significantly reduced biofilm formation, protease production and motility of *Escherichia coli*, whilst Jailani^48^ revealed that tannic acid inhibited the growth and biofilm formation of *A. tumefaciens*. Furthermore, tannic acid reduced the virulence features of *A. tumefaciens*, which are motility, exopolysaccharide production, protease production and cell surface hydrophobicity.

*Thevetia* sp. extract was less effective against both tested isolates, which agree with^49^ who proved that *Thevetia peruviana* leaves extract showed moderate anti-microbial activity against *Staphylococcus aureus*, Vice versa, Chauhan^50^ proved that *Thevetia peruviana* ethanol extract had greatest antibacterial effectiveness against *Escherichia coli*. Kumar^13^ exhibited that the tested extracts of *Thevetia peruviana* showed excellent inhibitory potential against *A. tumefaciens*. *Thevetia peruviana* extracts have shown inhibitory effects against various bacterial strains, including both Gram-positive and negative bacteria^51^. This general antibacterial activity suggests a potential for *Thevetia* extracts to affect *Agrobacterium* as well. The effective phytochemicals in *Thevetia* extract that may responsible for the antimicrobial activity include cardiac glycosides, saponins, flavonoids, phenols, and alkaloids^52,53^. These phytochemicals can exert their effects through various mechanisms, such as disrupting bacterial cell membranes, inhibiting enzyme activity, or interfering with bacterial DNA replication and protein synthesis^53^.

In a previous investigation, tomato treated with DL-aspartic, citric acid and L-arginine were minimized the root galls by 95.6, 94.3 and 93.02%, respectively. Meanwhile, the soil population of *M. javanica* were suppressed by 95.2, 92.9 and 92.6% with citric acid, L-arginine and gibberellic acid, respectively^7^. Citric and ascorbic acids were significantly stimulated the levels of total sugar, non-reduced sugar, total phenol, free phenol and proline moderately in tomato plants. The activation in tomato growth parameters may be attributed to the potentiality of treatments including organic acids to reduce the nematode infection on the root and/or increasing the resistance against plant nematode infestation^54,55^.

It was reported that plants treated with inducers may loss the juveniles of nematodes the ability to develop, in addition to find penetrate and settlement of the feeding site^56,57^. Also, using of β-amino butyric acid and ascorbic acid incited the levels of polyphenol oxidase (PPO) and peroxidase as well as total soluble phenol and proteins significantly at rang of 53.75 to 176.54% in tomato plants^8^. In a study by^58^ reported that using organic acids such as acetic acid, lactic acid, and their mixtures have nematicidal activity against *M. incognita* under laboratory conditions. Moreover, the root galls of chili pepper plants were decreased significantly within a range of 57.1 to 100% with single or mixture of acetic and lactic acids. In the same time the applied nematicide (Fosthiazate) achieved a 100% reduction with recommended and double dose.

The present results are also in accordance with^59^ who reported that ascorbic acid was more efficient than citric acid in reducing root galls and egg masses of *M. incognita* in Okra plants. Oxamyl reduced galls (96.90%) and egg masses (99.10%) significantly. The application of ascorbic, acetylsalicylic and salicylic acids as soil drench pre-inoculation was more effective in reducing the nematode population than at or post inoculation time^60^. Ascorbic acid application suppressed the soil population and development of *M. javanica* in the potato crop^61^ and the sugar beet crop^62^. Ascorbic acid and β-amino butyric acid elucidated significant reductions in egg masses, root galls and soil population of *M. incognita* on tomato plants^8^. Also, application of ascorbic acid as foliar spray at 1000 µg/l suppressed the presence of *M. incognita* infected soybean plants^63^.

In our study the extract of *Thevetia* sp. showed moderate bactericidal and nematicidal efficacy. But unfortunately, the provided litterateurs for nematicide or bactericide activity were very lack. However, *Thevetia* sp. extracts have insecticidal, fungicidal, bactericidal and rodenticidal performance^12^. Kumar^13^ revealed that different extracts of *Thevetia* sp. contain terpenoids, phenols, flavonoids, anthraquinones and free amino acids, whose toxic against varied list of microorganisms. Using the leaf extracts of *Thevetia peruviana* as bare-root dip treatment against in tomato and eggplant was reduced the development of the root-knot nematode (*Meloidogyne* sp.). the reduction effect was increased with the concentration of leaf extracts and dip duration^64^. This suggests that the active compounds within *Thevetia* extracts possess direct toxic effects on different life stages of the nematode. Rao^52^ revealed that the extracts of *Thevetia peruviana* have nematicidal effects which may have attributed to their bioactive compounds such as phenols, alkaloids and glycosides (e.g., thevetin, peruvoside, neriifolin). These compounds may disrupt the nematode’s physiological processes, interfere with its nervous system, or damage its cuticle, leading to paralysis, immobility, and ultimately death.

According to our data ascorbic acid showed improving in guava seedling growth which may attributed to the increment in photosynthetic rates and water use efficiency^65^. Also, ascorbic acid plays a vital role in regulating root growth by enhancing nutrient uptake and reducing stress in the soil rhizosphere^66^. In a recent study on guava seedlings under saline stress has shown that foliar application of ascorbic acid significantly boosts growth by improving stomatal conductance and gas exchange^67^. In the same context, citric acid showed improving in guava seedling growth by acts as a chelating agent, increasing the bioavailability of micronutrients like iron and phosphorus particularly in alkaline soils and enhanced chlorophyll content^68^. There aren’t direct known reasons can clarify why the methanolic extract of *Thevetia* sp. gave improve in the growth of guava seedlings. But it may be attributed to indirect effect by reducing the parasitic nematodes load on the roots due to the presence of toxic compounds or inhibited the growth of certain pathogenic microorganisms in soil^69^.

The GC-MS analysis of *Thevetia* sp. extract in our study is in parallel with^70^ who mentioned that the extract of *Thevetia* sp. contains trilinolein that has antibacterial, antinematodal and antifungal properties. Also, he stated that Hexadecanoic acid has antimicrobial, antioxidant and antifungal. *Thevetia* sp. included dodecanoic acid,2,3-bis (acetyloxy) propyl ester which utilized as an antifungal and antibacterial agent^71^. In our study, the active ingredient as cis-13-eicosenoic acid was exhibited antioxidant and antifungal activity^72^. In addition, 9,12,15-Octadecadienoic acid and Ethyl tridecanoate have antimicrobial and antioxidant effects^73,74^. Tetradecanoic acid exhibited antibacterial and antinematodal activities^75^.

## Conclusion

In this investigation we try to suggest an effective and eco-friendly solution to the problem of single or dual infections of *A. tumefaciens* and *M. incognita* which is one of the hardest problem that facing us in Egypt. The obtained results exhibited that the ascorbic acid has a reasonable potential for controlling the crown gall disease (*A. tumefaciens*) and the root-knot nematode (*M. incognita*). The extract of *Thevetia* sp. was the least effective treatment against both pathogens separately or in combination. Ascorbic acid, citric acid and the plant extract significantly induced the total phenol and proteins in guava seedlings in the presence of *A. tumefaciens* and *M. incognita* or their dual infection. Moreover, the plant growth indices were augmented with ascorbic and citric acids. Further studies are still needed.

## Data Availability

The datasets generated and analysed during the current study are available in the NCBI repository and GenBank under the accession number PP646500.
